# Nutrition Assessment and Counseling in Integrative Cancer Care: Effects on Patient Self-Reported Symptoms

**DOI:** 10.1177/15347354251342756

**Published:** 2025-05-24

**Authors:** Meroë Morse, Aimee J. Christie, Andrew Cusimano, Chandler Nguyen, Richard Wagner, Santhosshi Narayanan, Gabriel Lopez

**Affiliations:** 1 The University of Texas MD Anderson Cancer Center, Houston, TX, USA; 2 University of Texas Health Science Center, McGovern Medical School, Houston, TX, USA

**Keywords:** nutrition, oncology, patient-reported outcomes, integrative

## Abstract

**Background:**

Nutrition guidance can affect treatment and survival outcomes for patients with cancer. The effect of a single nutrition consult on patient-reported outcomes is not well known. This study describes characteristics of patients referred for a single outpatient nutrition consult in an integrative oncology center at a cancer hospital, examining effects on self-reported symptoms.

**Methods:**

We completed a retrospective chart review of 1517 adult oncology patients who completed a single nutrition consult. Demographics, clinical characteristics, and reasons for referral were extracted. Modified Edmonton Symptom Assessment System (mESAS) and Patient-Reported Outcomes Measurement Information System (PROMIS10) scores were collected. Standard descriptive statistics and the Wilcoxon signed-rank test were used.

**Results:**

Most patients were white, women, overweight/obese with breast cancer and non-advanced disease, mean age 55.3 years. Primary reasons for referral were “lifestyle change,” “overweight weight-related,” and “nutrition knowledge deficit.” PROMIS10 results were average. Other than sleep and hot flashes, mESAS symptom scores at baseline were in the mild range. All change scores after nutrition consult were statistically but not clinically significant.

**Conclusion:**

Patients presenting for nutrition counseling had a mild symptom burden at baseline and experienced statistically, not clinically, significant improvement in self-reported symptoms following a single visit. Additional research is needed to explore strategies to enhance the intervention and understand the effects of multiple follow-up sessions.

## Plain Language Summary

This study looked at how a single nutrition consultation affects cancer patients. Records of 1517 adult cancer patients who had at least one nutrition consultation were reviewed. Most patients were white women who were overweight/obese and had breast cancer. The main reasons for the consultation were to make lifestyle changes, manage weight, and improve nutrition knowledge. The study found that after the consultation, patients reported some improvement in their symptoms, but one consultation was not enough to make a significant difference in their overall well-being. The researchers suggested that more research is needed to understand the long-term effects of nutrition counseling on cancer patients.

## Introduction

An integrative approach to cancer care emphasizes the importance of diet quality and food synergy.^1^ Leading oncology organizations guide cancer patients and survivors to follow a diet high in vegetables, fruits, whole grains and low in processed foods, saturated fat, red meat, and alcohol (see Table 1).^2^ These recommendations are based on the evidence that high-quality eating patterns impact treatment response, tumor growth, and survival outcomes in patients with cancer.^3,4^ Historically, nutrition counseling in oncology patients occurred in the context of weight loss, cancer cachexia, and the management of cancer or treatment-related side effects.^5-7^ There is increasing evidence supporting the role of clinical nutrition interventions in decreasing therapy toxicity, dose modifications, and hospitalizations^8^ while improving treatment adherence,^9-11^ relative-dose intensity, and survival outcomes.^12,13^

**Table 1.** AICR/World Cancer Research Fund International Recommendations for Health Promotion.

• Be as lean as possible without becoming underweight • Be physically active for at least 30 min every day; limit sedentary habits • Eat a high-quality diet rich in whole grains, vegetables, fruits, and beans • Limit consumption of “fast foods” and other processed foods high in fat, starches, or sugars • Limit consumption of red and processed meat, sugar-sweetened drinks • Limit alcohol consumption • Do not use supplements for cancer prevention • After a cancer diagnosis: follow our recommendations when possible

Source: Clinton et al.^2^

Nutrition recommendations are a core aspect of the integrative approach to cancer care, yet nutritional counseling is often overlooked in cancer patients with no food or appetite-related symptoms. An estimated 80% of cancer patients are malnourished,^14^ yet fewer than 60% of those patients receive nutrition support, in part due to limited access to oncology-trained dietitians.^5^ Research also has shown obesity is a poor prognostic factor for patients with breast cancer, resulting in increased risk of cancer-associated mortality and all-cause mortality.^15^ A report by Trujillo et al estimated that for each oncology dietitian, there is an average of 2308 oncology patients.^16^ Maintaining a healthy diet and body composition is essential during and after cancer treatment. After treatment, eating a plant-centered diet, engaging in physical activity, and maintaining a healthy body composition can reduce the risk of cancer recurrence, secondary cancers, and other non-communicable diseases and improve overall survival.^17-21^ Diet quality after cancer diagnosis is also known to contribute to cancer survival. In a 2018 analysis of women with breast cancer, researchers found that poor diet quality after diagnosis was associated with a higher risk of death from breast cancer.^22^ These findings are consistent with the results from the Physicians’ Health Study of more than 22 000 male physicians, in which men diagnosed with prostate cancer and consuming a Western eating pattern (high in fat, sugar, cholesterol, and sodium) had a 2.5-fold higher risk of prostate cancer-specific death.^23^

The Integrative Medicine Center (IMC) at UT MD Anderson Cancer Center follows the established integrative oncology aims to optimize health, quality of life, and clinical outcomes.^24^ A multidisciplinary team of physicians, nurses, dietitians, physical therapists, health psychologists, acupuncturists, yoga therapists, and more are utilized to help address the physical, mind-body, and social aspects of health. Nutrition counseling, led by a registered dietitian, provides evidence-informed recommendations, and personalized feedback. Referrals are made to nutrition counseling after the patient is first evaluated by an integrative physician and/or advanced practice provider as part of a comprehensive evaluation of the patient’s physical and psychosocial needs.^25^ An integrative care plan may consist of a nutrition consult (one-to-one setting) for patients to incorporate dietary recommendations before, during, and/or after cancer treatment.

Nutrition counseling is defined as “advising and assisting individuals and groups on appropriate nutrition intake by integrating information from the nutrition assessment with information on food and other sources of nutrients and meal preparation consistent with cultural background and socioeconomic status.”^26^ Dietitians are qualified to assess an individual’s nutrition and health needs and consider the safety precautions and contraindications to improve the patient’s overall wellbeing. The IMC follows nutrition guidelines provided by the American Institute of Cancer Research (AICR) and World Cancer Research Fund International (WCRFI), as well as systematic literature reviews on diet, physical activity, and cancer risk.^2^ The American Cancer Society has similar guidelines^27^ on nutrition and physical activity for cancer prevention, which, along with the 2020 to 2025 USDA Dietary Guidelines for Americans, are designed to promote health, reduce the risk of chronic disease, and reduce the prevalence of overweight and obesity.

The aims of this study are to describe (1) the characteristics of patients who completed an integrative nutrition consultation and (2) changes in patient-reported symptom burden following a single nutrition consult. Within our integrative nutrition counseling service model, we routinely implement self-reported outcome assessments into clinical care to objectively monitor interventions’ immediate and long-term effects. This retrospective analysis reports on demographic, medical, and psychosocial characteristics of patients referred for nutrition counseling at a comprehensive cancer center and examines the effects of a single nutrition consultation on self-reported symptoms. Understanding patients’ responses to one nutrition counseling session will better inform integrative oncology practices and opportunities for intervention and innovation.

## Methods

### Participants

A retrospective chart review was conducted of consecutive patients who completed a nutrition consultation appointment within the IMC between January 27, 2017 and January 27, 2022, which included 1517 unique patients. Only patients ≥18 years of age and with a cancer diagnosis were eligible for the review. Patients were at various stages of the disease trajectory, including during cancer treatment, on active surveillance, and in the survivorship stage. All questionnaire data collection was part of an institutional review board-approved protocol (DR11-0149, informed consent waiver) and stored in a secured, HIPAA-compliant FileMaker Pro database.

### Intervention

#### Measures

##### Demographics and Clinical Characteristics

As part of the standard of care, patients being seen for their first outpatient nutrition consultation were asked to complete a series of assessments. Demographics (age, sex, race, ethnicity, marital status, employment) and clinical characteristics (cancer diagnosis and stage, body mass index—[kg/cm^2^] × 10 000) were extracted from the electronic medical record using a web intelligence tool. Reasons for referral (see Table 3 for list) were also collected, as documented by the referring integrative oncology physician or advanced practice provider. Reasons for referral are listed as orders in electronic medical records, and multiple reasons can be selected by the providers.

##### Modified Edmonton Symptom Assessment System (mESAS, 16-Item)

The original ESAS scale was developed in 1991 to measure physical and psychological symptom burden in supportive and palliative care patients.^28^ Patients rate symptom intensity over the previous 24 hours using a visual numeric scale of 0 (no symptom) to 10 (worst possible symptom). The original scale included 10 symptoms: pain, fatigue, nausea, depression, anxiety, drowsiness, loss of appetite, decreased sense of wellbeing, shortness of breath, and sleep. Generally, mESAS scores of 1 to 3 are considered mild, 4 to 6 as moderate, and 7 to 10 as severe. In 2012 and 2015, the ESAS was modified to include 6 additional items particularly relevant for integrative medicine patients: spiritual distress, financial distress, numbness/tingling, hot flashes, dry mouth, and memory.^29,30^ Pre- and post-ESAS scores were collected and recorded immediately before and after the nutrition consultations.

The ESAS includes subscales supported by cluster analysis.^31,32^ The Global Distress Score (GDS) is the sum of 9 core items, including pain, fatigue, nausea, depression, anxiety, drowsiness, appetite, sense of well-being, and shortness of breath (total score 0-90). The Physical Symptom Score (PSS) is the sum of pain, fatigue, nausea, drowsiness, appetite, and shortness of breath scores (total 0-60). The Psychological Distress Score (PDS) is the sum of depression and anxiety scores (total 0-20). A clinically significant difference (change) is ≥1 point for all individual symptoms.^33^ For predetermined subscales, clinically significant improvement is defined as differences ≥3 for the Physical Symptom Score, ≥2 for the Psychological Distress Score, and ≥3 for the Global Distress Score.^34^

##### Patient-Reported Outcomes Measurement Information System (PROMIS10)

The PROMIS10 is a general assessment of the global health-related quality of life, including 10 self-report items.^35^ Responses are on a 5-point scale (ie, poor, fair, good, very good, excellent), and 1 item is on an 11-point scale. Items are summed to create the subscales of global mental health and global physical health. Lower scores represent worse mental or physical health. Responses are converted into a T-score metric, with T-score distributions standardized to the US population. A T-score of 50 reflects the general population mean, and scores between 40 and 60 are within 1 standard deviation of the mean and considered average. Scores above 60 are considered high and better than average; scores below 40 are considered low and worse than average. Patients complete the PROMIS10 during the initial integrative oncology consultation.

### Statistical Analysis

Data were summarized using standard descriptive statistics such as mean, standard deviation, median, and range for continuous variables and frequency and proportion for categorical variables. Reasons for referral, PROMIS10 subscores, and mESAS scores were summarized using descriptive statistics. Data from the mESAS were not normally distributed; thus, the Wilcoxon signed-rank test was applied to examine changes pre-and post-consultation. For calculations regarding symptom score change, only those reporting an ESAS symptom score ≥1 at baseline were included in the analysis. This exploratory study included multiple analyses, so the alpha level was reduced to a more conservative value (.01) to lower the type I error rate.

## Results

Table 2 summarizes the demographic and clinical characteristics of all patients referred. The majority were women (83.9%), white (69.3%), and approximately one-third (38.5%) were employed full-time, with mean age 55.3 years. About two-thirds of the sample had a body mass index (BMI) in the overweight or obese range (70.6%). The most common diagnosis was breast cancer (58.1%), and about a third of the sample had advanced disease (stage III or IV, 37.4%).

**Table 2.** Characteristics of Patients Attending an Initial Integrative Oncology Nutrition Consultation.

Characteristic	n (%) n = 1517
Age mean (SD)	55.3 (12.8)
Sex	
Female	1273 (83.9)
Male	244 (16.1)
Race	
White	1052 (69.3)
African American	227 (15.0)
Asian	85 (5.6)
Other	153 (10.1)
Ethnicity	
Non-Hispanic	1251 (82.5)
Hispanic or Latino	227 (15.0)
Unknown	39 (2.6)
Marital status	
Married/significant other	1080 (71.2)
Single	203 (13.4)
Divorced/legally separated	167 (11.0)
Widowed	55 (3.6)
Unknown	12 (0.8)
Employment	
Full time	584 (38.5)
Retired	294 (19.4)
Not employed/disabled	300 (19.8)
Self-employed/part-time/student	166 (10.9)
Unknown/missing	173 (11.4)
BMI	
Obese (BMI ≥ 30)	638 (42.1)
Overweight (BMI 25.0-29.9)	433 (28.5)
Normal weight (BMI 18.5-24.9)	414 (27.3)
Underweight (BMI < 18.5)	32 (2.1)
Cancer diagnosis	
Breast	882 (58.1)
Gynecologic	109 (7.2)
Genitourinary	108 (7.1)
Gastrointestinal	98 (6.5)
Lymphoma/myeloma	69 (4.5)
Central nervous system/neurologic	43 (2.8)
Thoracic	43 (2.8)
Endocrine	38 (2.5)
Head and neck	34 (2.2)
Leukemia	30 (2.0)
Skin/melanoma	23 (1.5)
Sarcoma	17 (1.1)
Other	12 (0.8)
Bone/Skeletal	7 (0.5)
Hematological	4 (0.3)
Cancer staging	
Stage 0	69 (4.5)
Stage I	416 (27.4)
Stage II	298 (19.6)
Stage III	289 (19.1)
Stage IV	277 (18.3)
Unknown, unstaged, no stage	168 (11.1)

Abbreviation: BMI, body mass index.

Table 3 represents the reasons for referral to nutrition by the ordering provider. The top 3 reasons (and percent of sample) were “lifestyle change” (62.6%), “overweight weight-related” (50.4%), and “nutrition knowledge deficit” (44.8%). Regarding global physical and mental health, PROMIS10 results show that mean t-scores for the sample were in the average range (Table 4).

**Table 3.** Physician Indicated Reasons for Referral to Integrative Oncology Nutrition Consult.

Reason for referral	Frequency n (%)
Lifestyle change	950 (62.6)
Overweight weight-related	765 (50.4)
Nutrition knowledge deficit	679 (44.8)
Inadequate nutrition intake	664 (43.8)
Obesity	655 (43.2)
Undesirable food choices	346 (22.8)
Nutrition counseling	287 (18.9)
Disordered eating	256 (16.9)
Overall health	9 (0.6)
Morbid obesity	6 (0.4)
Related to treatment	6 (0.04)
Related to radiation	4 (0.3)
Related to chemo	3 (0.2)

Multiple reasons can be selected for each patient.

**Table 4.** PROMIS10 Scores.

PROMIS10	Mean (SD)	T-score	Min–Max T-scores
Global Physical Health	13.6 (2.8)	44.3 (7.9)	19.9-67.7
Global Mental Health	13.2 (3.1)	46.5 (8.0)	21.2-67.6

Missing n = 212 for physical health and n = 208 for mental health.

Table 5 shows the results of the mESAS scores among patients with individual symptom scores of 1 or greater (or 2 or greater for PSS and 3 or greater for PHS and GDS) before the nutrition consultation. Regarding missing data, 6.4% of the sample (n = 97) did not complete mESAS pre-assessment, and 14.5% (n = 220) did not complete the mESAS post-assessment. Prior to the nutrition consultation, most mESAS symptom scores were in the mild (1-3) range. Symptoms that were in the moderate range included sleep and hot flashes. All change scores were statistically significant but not clinically significant.

**Table 5.** Patient Reported Symptoms Before and After Initial Integrative Oncology Nutrition Consultation.

ESAS symptom	n	Mean (SD), pre	Mean (SD), post	Significance test
Pain	862	3.24 (2.15)	2.88 (2.27)	*z* = −8.07, *P* < .001
Fatigue	1097	3.70 (2.26)	3.12 (2.28)	*z* = −12.38, *P* < .001
Nausea	258	2.60 (1.99)	2.16 (2.00)	*z* = −4.94, *P* < .001
Depression	578	3.20 (2.09)	2.76 (2.21)	*z* = −7.89, *P* < .001
Anxiety	899	3.19 (2.11)	2.62 (2.11)	*z* = −10.36, *P* < .001
Drowsiness	703	3.02 (2.02)	2.61 (2.17)	*z* = −6.91, *P* < .001
Shortness of breath	361	2.80 (2.07)	2.17 (2.03)	*z* = −7.60, *P* < .001
Appetite	838	3.81 (2.25)	3.42 (2.30)	*z* = −5.73, *P* < .001
Well-being	1067	3.63 (2.07)	3.00 (2.19)	*z* = −12.13, *P* < .001
Sleep	1133	4.36 (2.42)	3.87 (2.57)	*z* = −10.92, *P* < .001
Financial distress	722	3.95 (2.51)	3.59 (2.65)	*z* = −7.84, *P* < .001
Spiritual pain	334	2.83 (2.05)	2.47 (2.13)	*z* = −5.52, *P* < .001
Hot flashes	626	4.23 (2.60)	3.61 (2.73)	*z* = −9.37, *P* < .001
Dry mouth	613	3.40 (2.33)	2.93 (2.38)	*z* = −7.50, *P* < .001
Memory	1031	3.68 (2.16)	3.33 (2.19)	*z* = −8.36, *P* < .001
Numbness/Tingling	724	3.79 (2.41)	3.31 (2.46)	*z* = −8.68, *P* < .001
Physical Symptom Score	1267	10.71 (8.26)	9.57 (7.93)	*z* = −10.27 *P* < .001
Psychological Distress Score	942	5.00 (3.91)	4.25 (3.89)	*z* = −10.33 *P* < .001
Global Distress Score	1318	16.57 (12.58)	14.54 (12.09)	*z* = −13.19 *P* < .001

Physical Symptom Score = Pain + Fatigue + Nausea + Drowsiness + Shortness of breath + Appetite (0-60).

Psychological Distress Score = Depression + Anxiety (0-20).

Global Distress Score = Pain + Fatigue + Nausea + Drowsiness + Shortness of breath + Well Being + Appetite + Depression + Anxiety (0-90).

## Discussion

Our study describes the characteristics of patients attending an outpatient integrative nutrition consultation, reasons for referral, and patient-reported outcomes of global health and symptom burden. In order to create a frame of reference, we can consider how the patients in the current nutrition consultation sample compare and contrast to patients attending their first IMC consultation with an integrative physician. The IMC consultation is the first evaluation completed by the integrative medicine team where a referral to nutrition would occur. We referred to a previously published paper on IMC consults of 1827 patients seen between 2019 and 2020.^36^ Compared to the IMC consult sample, the current nutrition consult sample had more women (83.9% vs 72.6%), but patients were of similar ages. The higher proportion of women may reflect research that shows women, compared to men, are more likely to engage in healthy lifestyle-related behavior change,^37,38^ more likely to be dieting and attach greater emphasis to healthy eating.^39^ Compared to the IMC consult sample, the nutrition consult sample had slightly greater racial and ethnic diversity, with 15% versus 10% of African Americans and 15% versus 12.8% of Hispanic or Latino patients. A higher proportion of patients referred for nutrition counseling had BMIs in the overweight or obese range, 70.6% versus 64.5%. This would be consistent with the goal of referral for healthy lifestyle changes and potential weight loss. The study sample reflects a range of cancer types and stages of treatment and disease, a notable strength consistent with the integrative approach to support all patients throughout their cancer trajectory.

More patients with breast cancer attended a nutrition consultation compared to those seen as IMC consults (58.1% vs 39.1%).^36^ There is consistent, high-quality evidence on the survival impact of diet, body fatness, and physical activity for female breast cancer patients.^40^ This growing evidence base emphasizes the importance of oncology team referrals for lifestyle support and creates a demand for the use of lifestyle-related screening tools and clinical referral algorithms. As a direct result of this effort and observed practice patterns,^41,42^ our institution has developed a *Breast Cancer Initiative* wherein a streamlined referral process now exists between the breast center and the IMC after screening for dietary and exercise habits. The Breast Cancer Initiative was established in 2019 to address the unmet need for healthy lifestyle guidance in newly diagnosed breast cancer patients. Lifestyle Screening questions assess 3 areas: (1) BMI; (2) amount of typical meal ie, plant-based; and (3) amount of weekly physical activity. If patients had BMI ≥25 and/or consumed <2/3 plant foods and/or performed <150 minutes physical activity a week, referrals were placed to the IMC for personalized oncology-specific healthy behavior support. The Lifestyle Screening questionnaire is embedded as a flowsheet in the electronic health record and clinic nurses are prompted every 4 months to re-screen patients. Over the course of 2 years, and screening more than 1000 patients, over 70% of patients met 1 or more of the screening criteria and were eligible for referral to IMC.^43^ The Lifestyle Screening questionnaire is a replicable and systematic approach to assess for healthy lifestyle measures and create an opportunity for referral to address and improve lifestyle behaviors during and after cancer treatment.

As expected, the top 3 reasons for referral were related to lifestyle modification and nutrition-based management of obesity, whereas less than 1% of patients were referred for “treatment-related nutrition issues.” In contrast to a medical nutrition referral, integrative nutrition consultations emphasize adherence to the AICR recommendations, supporting nutrition-based strategies with effects on cancer prevention, recurrence, and survivorship. This is consistent with a personalized integrative oncology treatment plan which aims to empower patients to identify and improve upon modifiable lifestyle factors to improve outcomes, symptoms burden, and quality of life.

This study has 2 patient reported outcomes, a measure of global physical and mental health and a measure of symptom burden. The measure of global physical and mental health as measured by the PROMIS10 showed mean *t*-scores within 10 points of 50, which is within the average range of this measure. The standard deviations dip below a t-score of 40, suggesting that this sample of patients attending a nutrition consultation are reporting global mental and physical in the low average range, or between “fair” and “good.”^44^ PROMIS10 results are similar to previous reports of IMC patients^36,45^ and reports in other studies of cancer patients showing mean *t*-scores in the average range.^46,47^ This highlights the role of integrative medicine regarding treating the whole person and focusing on overall well-being during treatment and throughout survivorship.

The second patient-reported outcome reflects symptom burden, as measured by the mESAS. Prior to the nutrition consultation, symptom scores were generally in the mild range with sleep and hot flashes scoring in the moderate range. Previous patient ESAS results in our clinic show similar mild scores.^25,36,41,42,45^ Other studies have also shown that patients in integrative medicine clinics endorse mild to moderate symptom burden.^48^ The mild symptom burden reported by the study patients could be partially explained by the fact that only 18.3% of the patients in our study had stage IV disease. Patients with severe symptom burden are likely referred to the supportive and palliative care center. Alternatively, patients with acute severe symptoms may have been directed to the dietitian assigned to them as part of their primary disease center. Despite the low overall baseline symptom burden, our analysis revealed that patients experienced statistically significant improvements in physical, psychological, and global distress following a single integrative nutrition consult. This retrospective study suggests that the act of engaging with an integrative practitioner, reviewing nutrition guidelines, and making personalized nutrition recommendations reduces subjective symptom burden. Reductions are small and change scores did not reach clinical significance. While not explicitly studied, we speculate that the patient-provider interaction offers a sense of empowerment, respect, and uses a shared decision-making approach to develop a dietary plan which patients can follow at home. The current study suggests that this engagement between the patient and provider may have effects beyond the specific indication for the visit. Further examination of these unintended but potentially beneficial effects is needed. Similar impacts on psychosocial symptoms, namely reduced anxiety and increased hope, following a single clinical encounter have been previously observed in other research settings.^49^ It is possible that tracking ESAS scores over several nutrition sessions may reflect more clinically meaningful change. A consult with an integrative dietitian provides a unique opportunity for patient education, empowerment, and symptom-management through lifestyle and diet modification.

While many cancer centers are beginning to incorporate integrative and lifestyle support into the treatment plan for their patients, to date, this is the first study assessing the effects of a single nutrition consult on physical and psychological distress scores in patients with cancer. Our findings parallel the findings of our center’s integrative treatment interventions in which statistically significant reductions in symptom burden were reported following a single encounter with an integrative practitioner, including: yoga via telehealth,^50^ oncology massage therapy,^51^ physical therapy,^52^ and oncology acupuncture therapy.^53^ Similar research that assessed the effects of integrative health and medicine modalities on outpatients found that multiple modalities yielded immediate and clinically meaningful reductions in symptoms.^54^

### Limitations

While results from this study showed improvement in a patient-reported outcome after a single nutrition consult, the “why” behind that improvement was not discerned. Furthermore, it is unclear if symptom relief is maintained or continues over time. This retrospective study of patients from a large urban comprehensive cancer center may not be generalizable to integrative oncology practices around the globe or in community settings. The patient population was a convenience sample of patients who attended the nutrition consultation session within the study timeframe. It is unclear how patients who attended may differ from those who did not follow through with the nutrition referral. Since this study reflects a retrospective review of our routine clinical care, it did not include more qualitative measures of the patient experience or perceptions of the nutrition consultation visit.

### Future Directions

Understanding the patient’s perspective regarding the role of nutrition in cancer outcomes, recurrence, and survival will help identify gaps and opportunities to incorporate nutrition interventions and support into conventional oncology practice. This can be accomplished through patient surveys assessing beliefs, knowledge, confidence, and motivations. Additionally, future research can examine whether a single nutrition consultation addresses concerns about food and eating behaviors. For example, including measures or items that assess stress eating or emotional eating may better reflect patient nutrition concerns. Alternatively, a motivational interviewing framework could be used to assess change over one session. Patients could be asked the importance of making dietary changes, their confidence in their ability to make dietary changes, and their readiness to make dietary changes pre and post nutrition consultation. Further efforts to increase referral streams and engagement of men in an integrative nutrition consult are warranted. Finally, it’s worth considering the importance of appropriately training dietitians to be prepared for the emotional and physical complexities seen in cancer patients, which may help to reduce burnout in these providers.^55^

## Conclusions

Our study describes the real-world, demographic, medical, and psychosocial characteristics of patients presenting to an integrative oncology nutrition appointment in a tertiary care cancer center. Similarly, it details the range of referral reasons for a single outpatient integrative nutrition consult and examines the effects this appointment had on self-reported symptoms. While the study was not designed to measure longitudinal changes in nutrition outcomes or body composition, we did note a statistically significant reduction in symptom burden from a single nutrition consult. Research is mounting regarding the clinical value of early nutrition screening and intervention at every stage of cancer care.^56-58^ There is still relatively poor awareness among clinicians, patients, and policy-makers that early and frequent nutrition interventions are a cornerstone for improving cancer survival outcomes.^59^ This study offers early indication that there may be potential benefits from a single nutrition consult on clinically relevant symptoms; however, we speculate that optimal outcomes in nutritional habits, body composition, and symptom burden will require repeated visits, close follow-up, and measurable dietary change. Nutrition consultation, irrespective of cancer and treatment stage, should be incorporated as standard of care to address both patients’ survival outcomes and quality of life. Furthermore, future research should emphasize implementation studies within oncology settings to identify best practices for nutrition screening, referral, and longitudinal care coordination.
